# Short-term outcomes of extracorporeal shock wave therapy for the treatment of chronic non-calcific tendinopathy of the supraspinatus: a double-blind, randomized, placebo-controlled trial

**DOI:** 10.1186/1471-2474-13-86

**Published:** 2012-06-06

**Authors:** Olimpio Galasso, Ernesto Amelio, Daria Anna Riccelli, Giorgio Gasparini

**Affiliations:** 1 Department of Orthopaedic and Trauma Surgery, Magna Græcia University, V.le Europa, 88100, Catanzaro, Italy; 2 Extracorporeal Shock Wave Research Unit, Policlinico G.B. Rossi, Largo LA Scuro 10, 37134, Verona, Italy

## Abstract

**Background:**

There is evidence supporting the use of extracorporeal shock wave therapy (ESWT) in calcific tendinopathy of the rotator cuff, but the best current evidence does not support its use in non-calcifying tendinopathy. We conducted a randomized placebo-controlled trial to investigate the efficacy and safety of low energy ESWT for non-calcifying tendinopathy of the rotator cuff.

**Methods:**

20 patients with non-calcifying supraspinatus tendinopathy (NCST) were randomized to an active or a sham treatment group. Physical, blood, roentgenographic, and MRI examinations of the shoulder were conducted to verify that patients met the inclusion and exclusion criteria. These examinations were repeated six and twelve weeks after treatments. Effectiveness was determined by comparison of the mean improvement in the Constant and Murley score (CMS) between the treatment and the placebo groups at three months. Safety was assessed by analyzing the number and severity of adverse events.

**Results:**

All the patients completed the investigation protocol. At the final follow-up, significant improvement in the total CMS score and most of the CMS subscales was observed in the ESWT group when compared to the baseline values. Significantly higher total CMS, and significantly higher scores for CMS pain and ROM were observed in the ESWT group when compared to the placebo. No serious adverse events were noted after ESWT.

**Conclusions:**

Patients suffering from NCST may benefit from low energy ESWT, at least in short-term. The application protocol of ESWT is likely to play a key-role in a successful treatment. Future investigations should be undertaken on the long-term effects of this technique for the treatment of NCST.

**Trial registration:**

Current Controlled Trials ISRCTN41236511

## Background

Supraspinatus tendinopathy is a common and disabling condition that becomes more prevalent after middle age [1,2]. There exist many forms of conservative treatment but evidence for their efficacy is not well established [3].

The shock wave is a single-impulse acoustic wave generated by an electromagnetic, electrohydraulic or piezoelectric source. The energy at the focal point is recorded in millijoules per area (mJ/mm^2^) and based on this value, shock waves are classified as low, medium, or high energy [4]. In the last 20 years extracorporeal shock wave therapy (ESWT) has been widely used to treat enthesopathies [5,6]. Trials have examined the effect of ESWT on plantar fasciitis [7], epicondilytis [8] and jumper’s knee [9]. Good evidence is available to support the use of extracorporeal shock wave therapy (ESWT) in calcific tendinopathy of the rotator cuff, but the best current evidence does not support its use in non-calcifying tendinopathy of the rotator cuff [10]. However, only a limited number of studies have reported on ESWT for non-calcifying supraspinatus tendinopathy (NCST) in the English literature [11-13].

We conducted a study to investigate the efficacy and safety of low energy ESWT in patients suffering from chronic NCST and compared it to placebo. Effectiveness was determined by comparison of the mean improvement in the Constant and Murley score (CMS) in the treatment and the placebo groups at three months. Roentgenographic and MRI changes of the shoulder both within and between the two groups were evaluated at follow-up. Safety was assessed by analyzing the number and severity of adverse events associated with use of the investigational treatment.

## Methods

### Patients

The study protocol was approved by the local Ethics Committee and the research was carried out in compliance with the Helsinki Declaration. Patients with NCST who had failed conservative treatments for a minimum of four months were evaluated for enrollment in the study. The prior regimens of conservative treatments included: administration of at least one subacromial steroid injection, one course of non-pharmacological therapy for at least 3 weeks, and one course of non-steroidal anti-inflammatory drugs or analgesics. The clinical criteria to diagnose the supraspinatus tendinopathy were a minimum six-month period of painful shoulder and pain on the Jobe [14] or full can tests [15]. The can test consists in evaluating the patient’s ability to resist downward pressure on the arms held at 90° elevation in the scapular plane and 45° external rotation [15]. A screening interview with physical and blood examinations, an X-ray and an MRI of the shoulder were conducted to ensure that the referred patients met the inclusion and exclusion criteria (Table 1) and were willing to participate to the study. Informed consent was obtained subsequently. The blood examination consisted of a complete blood count, prothrombin time, partial thromboplastin time, and a pregnancy test for women of child-bearing potential. A general physical examination and CMS [16,17] were measured by an orthopedic surgeon blinded with respect to the treatment regimen of each patient. The CMS combines subjective and objective measurements in one score. The objective parameters (65 points) include the patient’s range of motion (ROM) and power, corresponding to the number of pounds of force recorded by the dynamometer. A static strength tester (CSD 300 Chatillon - Ametek Inc., U.S.A.) was used. The subjective parameters included pain and impact on activities of daily living (ADL), including positioning (35 points). The CMS increases as pain decreases and shoulder mobility increases, therefore the higher the CMS, the greater the improvement in the condition and quality of life of the patient. The CMS has been extensively validated and shows good intra- and inter-observer reproducibility [16-18].

**Table 1:** Inclusion and exclusion criteria

**Inclusion Criteria**	**Exclusion criteria**
1. Male and non-pregnant female patients 18 years of age or older (women of child-bearing potential must have a negative serum pregnancy test performed within 1-14 days prior to the treatment procedure) suffering from NCST as diagnosed by X-ray, MRI and physical examination.	1. Patient has a history of uncontrolled severe hypertension (systolic pressure > 180 mmHg, diastolic pressure > 110 mmHg).
2. Patient has not responded to a standard course of non-pharmacological and non-surgical conservative treatment for a minimum of three weeks. The treatment above consists of: therapeutic exercise, and/or ultrasound, and/or iontophoresis, and/or cryotherapy, and/or immobilization or activity modification.	2. Patient has unstable or uncontrolled angina, uncontrolled heart failure, or serious uncontrolled ventricular arrhythmias.
3. Patient has not responded to pharmacological treatment (one course of the standard dose of prescribed analgesic or NSAID) and has had at least one subacromial steroid injection.	3. Patient has a white blood cell count less than 2,000 or greater than 15,000, or platelet count less than 50,000.
4. Diagnosis of supraspinatus tendinopathy is only in one shoulder.	4. Patient has a known bleeding disorder or is currently being treated with anticoagulant therapy.
5. Patient has free passive range of movement and at least 90 degrees active abduction in the affected shoulder.	5. Patient is currently being treated with a narcotic or NSAIDs and/or has used analgesics or NSAIDs within the 72 hours prior to the SV.
6. Patient is willing to participate in the study and return for all scheduled follow-up visits.	6. Patient has participated in any other shoulder pain treatment research study within 30 days prior to the SV.
7. Patient is capable of giving, and has given, written informed consent.	7. Patient had prior shoulder surgery
	8. Patient received prior ESWT for any disease.
	9. Patient is complaining of pain in both shoulders.
	10. Patient has malignant tumors, irrespective of location.
	11. Patient has a cardiac pacemaker implant.
	12. Patient has anatomy that prevents the focusing of the device into the shoulder in the area of the supraspinatus tendon (e.g., extensive scarring, misalignment of previous fractures, non-unions or delayed fracture healing, congenital malformation, etc.).
	13. Patient has any upper extremity neurological disorder as diagnosed from focused neurological exam and neurophysiological studies (e.g. thoracic outlet syndrome, reflex sympathetic dystrophy, etc.).
	14. Patient has a full-thickness rotator cuff tear of any of the rotator cuff tendons as seen on MRI.
	15. Patient has an acromiohumeral interval less than 7mm as measured on a standard AP X-ray, or severe symptomatic degenerative changes in the glenohumeral or acromioclavicular joint.
	16. Patient has acute subacromial bursitis as diagnosed by MRI
	17. Patient has generalized polyarthritis, rheumatoid arthritis.
	18. Patient is allergic to local anaesthetic.

SV, indicates screening visit; NCST, non-calcific supraspinatus tendinopathy; AP, Anteroposterior; NSAIDs, Non-Steroidal Anti-Inflammatory Drugs.

Patients were considered a treatment success if they showed an improvement of at least 30 points, or their CMS at the study’s endpoint was at least 80% of the standard age- and gender-related value [11,19]. Patients observed a pain medication-free interval 3 days prior to each CMS evaluation. Baseline social, anthropometric, educational, and occupational variables that might be associated with the outcome were gathered through a study-specific questionnaire. After treatment and during follow-up, patients were restricted to the use a 1000 mg of acetaminophen per day in cases of pain, in order to facilitate comparison of the medications and usage among the patients and groups and across all follow-up visits. The patients were randomized to an active or sham treatment group using stratified random permuted blocks with an allocation ratio of 1:1 and they were unaware whether they had received treatment. One patient initially assigned to the placebo group was lost after randomization, thus leaving twenty individuals available for the study (i.e. 11 in the ESWT and 9 in the placebo group). The study did not allow for crossover. The cohorts were scheduled at different times to ensure that the individuals within the cohorts did not contact each other.

### Imaging studies

The X-ray exams consisted of anteroposterior and supraspinatus outlet views. Magnetic resonance imaging scans (Siemens Magnetom Synphony-Maestro-Class, 1,5T) were acquired for all patients and included: a fast spin-echo intermediate-weighted axial sequence, a fast spin-echo coronal oblique intermediate-weighted sequence, and coronal oblique and sagittal oblique fast spin-echo T2-weighted acquisitions with fat suppression. A supraspinatus tendinopathy was diagnosed if intermediate-weighted and T2-weighted images showed diffuse mildly increased signal intensity (not equal to that of fluid) and an intact tendon was observed [20]. A full-thickness tear was defined as a high T2 signal extending through the depth of the tendon [21]. X-rays and MRI studies were independently evaluated by two musculoskeletal radiologists, who were unaware of the clinical characteristics of the patients, and the same measurements were repeated twice on two separate days. Cohen’s kappa coefficient for inter-observer and intra-observer reliability of scoring was 0.78 and 0.80, respectively. A consensus decision on the scores was reached in a final common readout.

### Interventions

The Modulith® SLK system (Storz Medical AG, Tagerwilen, Switzerland) was the electromagnetic therapy source used to treat the patients. Localization and targeting were achieved by means of an in-line 7.5 MHz ultrasound transducer with a scanning depth range of 3-15 cm, located in the center of the therapy source. Shock waves were focused at an area 1 cm proximal to the insertion of the tendon in the bone, with the patient in a supine position. The treatment regimen required administration of two treatment sessions, each consisting of 3000 shockwaves at an energy flux density of 0.068 mJ/mm^2^, separated by a 7-day interval. A similar protocol showed to be effective in the treatment of calcific shoulder tendinopathy [22]. The sham treatment entailed use of the same device in which the shockwave generator was disconnected. A compact disc player with a prerecorded sound of the ramp-up shocks produced the sound characteristic of the device as if it had been normally activated. The speakers were stored under the upper cover of the shock wave generator. As shockwaves may cause pain and discomfort, patients in both groups received a subcutaneous injection of 2cc of 2% lidocaine above the subacromial space of the affected shoulder prior to each treatment. The patient was treated by an unblinded investigator not involved in the enrollment of the patients, their randomization, or their follow-up. The heart rate, blood pressure, body temperature, and respiration rate were measured before and immediately after each treatment. Treatments were performed as outpatient procedures.

Patients repeated the physical and blood examination and the CMS evaluation at both 6 and 12 weeks follow-up. At the latter follow-up the imaging studies were repeated. The use of shoulder pain medication or any other drug was recorded at the final treatment and during the follow-up period. Adverse effects were assessed by clinical examination and by a patient questionnaire directly after the ESWT/sham procedure and at every follow-up visit. An anticipated adverse device effect (Anticipated adverse event for ESWT) was considered an adverse event that had been previously identified as occurring with some frequency as a result of the device use; conversely, an unanticipated adverse device effect was one that had not been identified in its nature, severity, or frequency in the literature. Adverse events were evaluated by the investigator blinded to patient assignment. The patient’s subjective opinion of the treatment received was noted at the study’s conclusion. All findings were recorded on standardized forms. At the final follow-up, the patients in the control group still complaining of symptoms of supraspinatus tendinopathy were offered the real ESWT, while those in the active treatment group were informed of further options.

### Anticipated adverse event for ESWT

Subcutaneous hematoma at treatment site

Petechiae at treatment site

Ecchymosis at treatment site

Increased pain in treated shoulder

Skin redness at treatment site

Bleeding

Swelling of treated shoulder

Skin irritation at treatment site

Migraine

Syncope

Nausea/Vomiting

Feeling Unwell/Dizziness

A telephone recall of the ESWT patients was carried out nine years after treatment to collect data about the number of patients who eventually progressed to surgical intervention or other treatment options. At the same time, the satisfaction with ESWT and willingness to undergo the treatment again was also recorded.

### Statistical analysis

Mean, standard deviation and range were reported for the continuous variables, whereas counts described the categorical variables. Because of the low expected frequencies, a Monte Carlo method or Fisher’s exact test was used for testing the significance of comparisons of the categorical variables between the ESWT group and the control subjects. An unpaired t-test was used to compare the means of quantitative variables between the groups.

Due to the non-normal distribution, non-parametric tests were used to compare the obtained shoulder ratings before and three months after starting the treatment, and between the ESWT and placebo groups. In particular, a Mann-Whitney U-test was used to assess the difference in scores distributions between the treatment and placebo groups, whereas a Wilcoxon test was used to compare the scores before and after the initiation of treatment. To calculate the power (1 – β error probability; two tailed) achieved by our statistical tests, we considered the actual sample size, the observed effect size, and the α value = 0.05.

SPSS (version 17.0, SPSS Inc., Chicago, USA) and G*Power (Institut fur Experimentelle Psychologie, Heinrich Heine Universitat, Dusseldorf, Germany) software were used for the statistical analyses.

## Results

All the patients completed the investigation protocol. The baseline characteristics of the study population are shown in Table 2. The demographic and all clinical data except the BMI did not differ between the ESWT group and the placebo group at baseline. No significant differences on the physical parameters were noted immediately after treatments within and between the two groups of the study. As shown in Table 3, at the earlier follow-up significant CMS changes were noted only in the ESWT group. The comparison between this group and the placebo group showed significant differences for the total CMS and the ROM subscale. At the final follow-up (Table 4), significant improvement in the total CMS and all the subscales (except power) was observed in the ESWT group when compared to the baseline values. In contrast, within the placebo group no statistically significant differences were observed with baseline. When the groups were compared, significantly higher total CMS and significantly higher scores for pain and ROM were observed in the ESWT group. The number and percentage of successful treatments according to the different study groups at the final follow-up are shown in Table 5. The mean relative improvement in the total CMS at three months was significantly higher in the active treatment group than in the control group (74.5% and 15.2% respectively, p = 0.014).

**Table 2:** Baseline characteristics of the patients

	**ESWT (n = 11)**	**PLACEBO (n = 9)**	**P value**
*Age (years)	50.7 ± 8.44 (38-64)	51.11 ± 13.26 (36-74)	0.938^§^
Sex			
Men	7	4	0.653^†^
Women	4	5	
*BMI	27.4 ± 1.04 (26-29.4)	24.4 ± 3.15 (19-29)	0.024^§^
*Duration of symptoms (months)	45.36 ± 34.33 (11-131)	61.22 ± 24.04 (34-97)	0.258^§^
Affected side			
Right	7	6	0.999^†^
Left	4	3	
Patients with physiotherapy	7	8	0.319^†^
Acromion slope			
Type I	8	7	0.999^†^
Type II	2	2	
Type III	1	0	

*The values are given as mean **±** standard deviation (range).

ESWT, extracorporeal shock wave therapy; BMI, body mass index; ST, supraspinatus tendon.

^§^ Unpaired t-test.

^†^ Monte Carlo or Fisher exact test.

^‡^ Mann-Whitney U-test.

**Table 3:** Comparison of shoulder ratings before and 6 weeks after ESWT/sham treatment in the study or placebo groups

**CMS**	**ESWT**	**PLACEBO**	**P value**^**1**^
PAIN			
Baseline	2.72 ± 2.61 (0-5)	3.33 ± 2.5 (0-5)	0.592
6 weeks	8.18 ± 3.37 (5-15)	4.44 ± 3.9 (0-10)	0.045
P value^2^	0.006	0.414	
ADL			
Baseline	10.27 ± 3.28 (5-18)	11.55 ± 4.21 (6-18)	0.378
6 weeks	15.1 ± 3.83 (7-20)	11 ± 5.48 (2-20)	0.068
P value^2^	0.01	0.674	
ROM			
Baseline	16.18 ± 4.68 (10-24)	16.67 ± 8.36 (2-26)	0.878
6 weeks	27.27 ± 8.5 (12-40)	17.1 ± 9.06 (8-32)	0.038
P value^2^	0.006	0.618	
POWER			
Baseline	13.27 ± 5.4 (5-20)	10.11 ± 3.18 (5-15)	0.170
6 weeks	13.36 ± 4.3 (7-18)	10.55 ± 4.21 (6-19)	0.174
P value^2^	0.834	0.726	
TOTAL			
Baseline	42.45 ± 9.83 (29-61)	41.67 ± 12.53 (20-57)	0.970
6 weeks	64 ± 16.6 (32-87)	43.11 ± 19.16 (18-70)	0.018
P value^2^	0.004	0.368	

The values are given as mean **±** standard deviation (range).

^1^ Comparison between treatment and control group both before and after treatment (Mann-Whitney U- test).

^2^ Comparison between before and after treatment within each group (Wilcoxon test).

ESWT, indicates extracorporeal shock wave therapy; ADL, activity of daily living; ROM, range of motion; CMS, Constant and Murley Score.

**Table 4:** Comparison of shoulder ratings before and 3 months after ESWT/sham treatment in the study and placebo groups

**CMS**	**ESWT**	**PLACEBO**	**P value**^**1**^
PAIN			
Baseline	2.72 ± 2.61 (0-5)	3.33 ± 2.5 (0-5)	0.592
3 months	10.9 ± 4.37 (5-15)	6.11 ± 4.86 (0-15)	0.039
P value^2^	0.004	0.096	
ADL			
Baseline	10.27 ± 3.28 (5-18)	11.55 ± 4.21 (6-18)	0.378
3 months	17 ± 4.22 (8-20)	12 ± 5.63 (4-20)	0.059
P value^2^	0.005	0.779	
ROM			
Baseline	16.18 ± 4.68 (10-24)	16.67 ± 8.36 (2-26)	0.878
3 months	30.9 ± 9.05 (16-40)	18.22 ± 10.50 (6-36)	0.012
P value^2^	0.005	0.635	
POWER			
Baseline	13.27 ± 5.40 (5-20)	10.11 ± 3.18 (5-15)	0.170
3 months	15.27 ± 6 (6-23)	11.67 ± 3.46 (6-16)	0.170
P value^2^	0.096	0.119	
TOTAL			
Baseline	42.45 ± 9.83 (29-61)	41.67 ± 12.53 (20-57)	0.970
3 months	74.09 ± 20.56 (39-98)	48 ± 22.3 (17-79)	0.023
P value^2^	0.003	0.260	

The values are given as mean **±** standard deviation (range).

^1^ Comparison between treatment and control group both before and after treatment (Mann-Whitney U- test).

^2^ Comparison between before and after treatment within each group (Wilcoxon test).

ESWT, indicates extracorporeal shock wave therapy; ADL, activity of daily living; ROM, range of motion; CMS, Constant and Murley Score.

**Table 5:** Success rate three months after shockwave therapy or sham treatment

** ESWT**				** PLACEBO**			
**PATIENT**	**CMS BASELINE**	**CMS at 3 MONTHS**	**SUCCESSFUL TREATMENT**	**PATIENT**	**CMS BASELINE**	**CMS at 3 MONTHS**	**SUCCESSFUL TREATMENT**
1	55	98	Yes	1	43	61	no
2	31	39	No	2	47	79	yes
3	45	94	Yes	3	54	78	yes
4	39	69	Yes	4	57	46	no
5	29	96	Yes	5	27	30	no
6	36	87	Yes	6	52	17	no
7	45	92	Yes	7	35	45	no
8	49	61	No	8	20	23	no
9	61	73	Yes	9	40	53	no
10	36	52	No				
11	41	55	No				
Successful Treatment			63,7%	Successful Treatment			22,3%

CMS, indicates Constant and Murley Score; ESWT, extracorporeal shock wave therapy.

No relevant adverse effects occurred during or after treatment, but there was a slight pain increase. Indeed, in the ESWT group, one patient reported a short-lived and bearable pain increase during the second session of therapy, while two patients reported an increase in pain at final follow-up. In the placebo group an increase was noted by only one patient, at three months after treatment.

As for the medications the patients used after treatment and during follow-up, the use of acetaminophen in the ESWT and placebo group averaged 0,73 ± 1,68 (range 0-5) and 6,78 ± 13,46 (range 0-41) days, respectively, but the difference was not statistically significant (p = 0.16). Seven out of eleven and five out of nine patients believed they had received active treatment in the ESWT and in the placebo group, respectively. The number of patients that the blinded investigator considered as actively treated were nine in the ESWT group and two in the placebo group.

The power analyses showed that the statistical tests, used to compare the subscale of ROM and the total CMS between ESWT and placebo groups at the final follow-up, had a power respectively of 75.5% and 70.3% to detect the observed differences. The statistical tests used to compare the CMS values at follow-up and baseline within the ESWT group had a power of 99.9% for the pain, 96.7% for the ADL, 99.1% for the ROM and 99.3% for the total score to detect the observed differences.

A telephone recall of the ESWT patients has been carried out nine years after treatment and we were able to collect data on 10 out of 11 individuals. No patient progressed to surgical intervention and two patients showed a recurrence of shoulder pain 3 and 4 years after ESWT, respectively. These patients were successfully treated with a second ESWT (1) and medication for pain with a regimen of scapulothoracic and glenohumeral range of motion and strengthening exercise (1). Nine years after ESWT all the patients available were satisfied with the treatment received and would have repeated the same therapy again.

## Discussion

Thousands of ESWT for NCST are currently performed in Europe [12], even if the available evidence does not support the use of this technique with this indication [10]. Because of the small number of studies on this topic and the few application protocols tested up to now [11-13], we re-evaluated the efficacy of low energy ESWT for NCST using a new protocol. Indeed, it has been clearly demonstrated that different protocols considerably modify the success rate of ESWT [22].

For the first time, we have demonstrated that patients suffering from NCST may benefit from ESWT. Our findings showed a significant CMS improvement in the ESWT but not in the control group six and twelve weeks after treatment. Furthermore, significant CMS differences between the groups at follow-up were also noted. The ESWT was found to be safe and well tolerated by the patients.

The best evidence for new treatments usually comes from randomized, placebo-controlled, double-blind studies and our work tried to provide compelling evidence that ESWT is effective in NCST. Our protocol included random sequence generation, allocation concealment, and blinding. A great effort was made to mask the real ESWT in order to eliminate subjective bias on the part of both experimental subjects and the experimenters. Indeed, the ESWT masking appeared less than optimal in previous similar studies [11,13]. Because the minimum effect size of the CMS is not known [23], we considered a clinically significant response a 30-point increase in the CMS [11] which is considerably higher than the values chosen by others to evaluate ESWT [22,23]. However, it should be noted that the 30-point difference it’s an arbitrary cut-off, not derived from research evidence. Interestingly, the study achieved a 100% rate of follow-up of patients, notwithstanding the presence of the placebo group.

Some limitations of the present investigation should also be acknowledged. The small sample size may have increased the risk of an underpowered randomized controlled trial. However, the differences in the CMS scores both within the ESWT group and between treatment groups at final follow-up were highly significant and the power analysis supported these findings, despite the small number of patients per group. Studies are considered to be adequately powered when there is about an 80% probability the study would show a treatment effect if it is present [24].

The short-term follow-up may have limited the generalizability of our study, even if the same follow-up interval was used previously in similar ESWT trials [11,23,25]. Notably, the short follow-up was useful to define the direct effects of ESWT on the clinical course of the condition and on the morphology of the supraspinatus tendon. Indeed, with a longer follow-up there might have been confusion between the effects of the treatment and spontaneous changes. A further restriction to increase the length of follow-up was the consideration that it is ethically and psychologically difficult to obtain informed consent to enter a study from patients presenting with chronic pain. The longer the duration of the study, the fewer are the individuals that would accept the possibility of receiving a sham treatment while suffering pain. Moreover, it should be noted that alternatives therapies are available to treat supraspinatus tendinopathy [3], and when treatments for a disorder already exist, it could be argued that it is unethical to create a placebo group that will receive no treatment at all. A different study design should be proposed to evaluate ESWT over a longer period.

The demonstration of ESWT efficacy in the short-term period is still a valuable finding of this investigation. Indeed, previous studies showed satisfactory outcomes in the short-term after other conservative therapies such as physical therapy [26] or subacromial cortisone injection [27-29]. However, we showed an higher improvement of CMS in comparison to the values reported by others treating the supraspinatus tendinopathy with ultrasounds or rehabilitation program with the same follow-up [26]. As for the corticosteroids, there is reasonably strong evidence that cortisone injection causes deleterious effects on the tendon and the outcomes deteriorates over time [30]. Indeed, the continued use of a local corticosteroid is discouraged [31]. On the contrary, no detrimental effects of ESWT for shoulder pain in the long-term period have been reported [32,33] and this treatment could eventually be repeated in case of recurrence of symptoms.

Our data are in keeping with the results of a recent study reporting significant increase in function and reduction of pain after low or high-energy ESWT in patients with NCST [12]. But our findings do not agree with the only two existing randomized, controlled studies that analyzed the efficacy of ESWT in NCST [11,13]. Indeed, Schmitt and colleagues reported significant CMS improvements both in low energy ESWT and in the placebo group three months after treatment, but no difference in CMS between the groups was noted [11], therefore shock waves were not recommended for NCST. However, in this study the method for administration of local anaesthetic involved use of a large bolus in the subacromial region (i.e. 10 cc of mepivacaine) and certain dosages of local anaesthetic are considered to be therapeutic [27]. Further weakness in the sham design and the method of assessment of the supraspinatus tendon with either MRI or ultrasounds must be considered. The trial by Speed [13] analyzed medium-energy ESWT in comparison with a placebo treatment for non-calcific tendinopathy of the rotator cuff, and confirmed the findings of Schmitt three months after the completion of therapies. However, some weakness appears also in this study due to the sham design. In the placebo group, the treatment head was deflated and contact with the skin was avoided, and no local anaesthesia was used. Since shockwaves may cause pain and discomfort, the ESWT masking here is less than optimal. Notwithstanding the limitations of these studies, any comparison with our findings is difficult because of the several variables that define the application parameters of ESWT. The shock wave generator, the number of impulses, the focusing of the shockwave with respect to the tendon insertion, the number and the interval between each treatment session, all are important factors that have to be carefully considered [6]. It is possible that different treatment regimens may be more effective than others [22] and, to our knowledge, the treatment protocol we used has not been utilized previously in a similar clinical setting.

Notably, to overcome at least in part the limitation of a short-term follow-up we performed a recall of patients nine years after treatments and an high satisfaction rate with treatment received together with a low recurrence of shoulder pain was noted.

We reported a successful treatment in 22% of our patients in the placebo group. This finding could be explained by the placebo effect rather than by the injection of a local anesthetic used to mask the treatment. Indeed, it is unlikely that a small dosage of local anesthetic into the subcutaneous fat of the shoulder would have a therapeutic effect. In fact, it was previously demonstrated that only higher dosages of this drug injected above the subacromial space are effective in the treatment of chronic rotator cuff tendinopathy [27]. It should be noted that the regression to the mean due to the spontaneous improvement or fluctuations in symptoms can lead to a false impression of the placebo effect [34].

One controlled prospective randomized trial on ESWT for calcifying tendinopathy of the rotator cuff has demonstrated that focusing the shock waves on the calcified area rather than on the insertion of the supraspinatus tendon is more effective [35], but no data are available regarding the best area to focus the shock waves in NCST. We focused the shock waves at an area one cm proximal to the insertion of the tendon in the bone where areas of avascularity have been described [36-38]. It has still not been determined whether vascular changes occur or are associated with rotator cuff pathology [39], however, experimental studies have demonstrated that shockwaves improve the blood supply to the tendon tissue throughout a neovascularization process [40] and low energy ESWT modulates the synthesis of nitric oxide [41,42], a molecule that plays a critical role in the regulation of vascular tone, angiogenesis [42], and in the degeneration of the tendon [43-45]. Recently, it has been suggested that shock waves behave fairly differently according to the clinical phase of the disease, even reducing the pathological angiogenesis associated with rotator cuff disease [46]. Further therapeutic mechanisms of ESWT in the treatment of tendinopathies have been hypothesized. ESWT have been shown to promote healing of tendinopathies by inducing TGF-beta1 and IGF-I [47]. However, the therapeutic mechanism of shock waves in the treatment of supraspinatus tendinopathy is still uncertain.

## Conclusion

ESWT was found to be safe and well-tolerated and, for the first time, it was demonstrated that patients suffering from NCST may benefit from low energy shock waves, at least in the short-term. The extracorporeal shock wave application protocol is likely to play a key-role in the successful treatment of NCST. Future investigations should be undertaken on the long-term effects of this technique in NCST.

## Competing interests

This study was supported, in part, by Storz Medical AG, Tagerwilen, Switzerland. The company put placebo shock wave equipment at our disposal but had no control over the data analysis and interpretation, the decision to publish, or the content of this article. The authors declare that they have no competing interests.

## Authors’ contributions

OG and EA, as principal investigators, conceived of this study and acquired the data and so had full access to all of the data in the study. DAR performed the statistical analyses. OG and DAR take responsibility for the accuracy of the data analyses, have interpreted the data and have written the manuscript. GG has made a critical revision of the manuscript for important intellectual content. All authors have read and approved the final manuscript.

## Pre-publication history

The pre-publication history for this paper can be accessed here:

http://www.biomedcentral.com/1471-2474/13/86/prepub
